# Impact of Perceived Product Value on Customer-Based Brand Equity: Marx’s Theory – Value-Based Perspective

**DOI:** 10.3389/fpsyg.2022.931064

**Published:** 2022-06-30

**Authors:** Yonggang Qiao, Xirui Yin, Gao Xing

**Affiliations:** ^1^School of Marxism, Suqian University, Suqian, China; ^2^School of Art and Media, Suqian University, Suqian, China; ^3^School of History and Culture, Harbin Normal University, Harbin, China

**Keywords:** product perceived value, customer-based brand equity, brand resonance, customer affective commitment, Marx’s theory

## Abstract

Management research is allocating energies to seek ways to improve organizational performance. Branding has become a significant phenomenon that academicians and scholars have studied. Improving the brand’s overall equity requires strategies that the brand managers must implement. Based on Marx’s theory, the present study attempts to determine the role of product perceived value on customer-based brand equity, brand resonance and customer affective commitment, respectively. Moreover, this study also tries to determine the mediating roles of brand resonance and customer affective commitment in the relationship between product perceived value and customer-based brand equity, respectively. For this purpose, the data were gathered from 310 customers of branding products in China. The present study applied partial least square structural equation modeling for empirical analyses using Smart PLS software. The present study’s findings acknowledge that product perceived value did not directly influence customer-based brand equity. However, results confirmed that product perceived value positively influences brand resonance and customer affective commitment. Furthermore, the outcomes of the present study also concluded that both brand resonance and affective commitment played a mediating role between product perceived value and customer-based brand equity, respectively. Theoretically, the study contributed to the literature by examining the influence of product perceived value on customer-based brand equity. The study also enriched the literature by providing key findings related to the mediating roles of brand resonance and customer affective commitment. Practically, the study is beneficial for the brands and they can enhance product perceived value by improving product design, effectively communicating product benefits, and executing effective promotional strategies.

## Introduction

Brands have played a significant role during the frenzy of acquisitions and mergers since the late 1980s. In the recent decade, brand valuation has shifted from developed economies to emerging economies which has changed the branding question considerably (Barros-Arrieta and García-Cali, 2021). In the present era of global marketing, branding strategy has become a significant indicator of the marketing mix and this factor is considered a key to achieving sustainable competitive advantage (Keller, 2021). Moreover, branding decisions have become a crucial factor for global marketing as the marketing managers are striving to capitalize the brand equity into reputable brands in the new economies. Globally, the efforts of the marketing managers can be seen in how they attract the attention of the customers and how well they build customer equity (Ruiz-Real et al., 2020).

Recently, studies in the discipline of customer-based brand equity have suggested a shift from measuring and conceptualizing the construct to examining the relationship with other concepts in the marketing domain (Surücü et al., 2019). Brand equity is a significant concept in the marketing-business practice and academic research as marketers can build strong brands and gain a competitive advantage (Keller, 2021). Customer-based brand equity is composed of five dimensions i.e., brand awareness, brand association, brand loyalty, perceived quality, and other proprietary assets (Cambra-Fierro et al., 2021). A brand’s customer-based brand equity is measured on the basis of how positively they react to a brand’s element of the marketing mix as compared to the same element of the marketing mix for other similar brands. Customer-based brand equity takes place when customers recognize the brand and possess strong, favorable, and unique perceptions about the brand. According to Hyun et al. (2022), customer-based brand equity is derived from brand value and brand strength. Brand value is gained through high future and current profits and brand strength is the association of the brand held by its customers. The confidence of the customers placed in the brand generated higher customer-based brand equity. This confidence allows the customers to become loyal and willing to pay a higher price for the brand products (Stocchi et al., 2020).

Brand equity is measured by three factors i.e., uniqueness, perceived quality/perceived value, and willingness of the customers to pay the high price (Tuncer et al., 2020). This suggests that perceived value is an integral component of customer-based brand quality. Product perceived value is one of the categories of consumer behavior. It is the evaluation or subjective judgment of the customers toward the product and the benefits received by buying the product (El-Adly, 2019). Product perceived value influences both purchase decisions and brand cognition of the customers. The perceived value is highly dependent on the benefits offered to the customer as the higher the benefits are, the more perceived value will be developed among the customers (Wang et al., 2019). Product perceive value is not related to the product price as cost-effective products can have higher perceived value if those products can solve customers’ problems effectively and quickly. In addition, there are mainly two dimensions of product perceived value i.e., perceived acquisition value which focuses on the gains received from the products. The other dimension of the perceived transaction value relates to the psychological pleasure or satisfaction of the customer after purchasing the product (Slack et al., 2020). Product perceived value is a significant construct in consumer behavior literature as it influences other consumer behavior aspects. For example, Chen and Lin (2019) suggested that perceived value directly impacts the repurchase intentions of the consumers. This indicates that perceived value is a factor that must be highly considered by the marketers and the organizations.

Brand resonance is referred to as the relationship between the brand and its customers including the willingness of the customers to buy and recommend the band to others (Duman et al., 2018). It is also how they perceive the value and goals of a particular brand. The experiences of the customers with the brand have the power to build a stronger brand resonance. The brand generally adds value to its products so that the consumers like the brand add develop repurchase intention toward the brand. Product evaluations developed by the brands help the customers to understand the brand, especially those products that are complex and manufactured by foreign companies not known to local consumers (Cheng et al., 2019). In other words, brand loyalty and equity are highly influenced by brand resonance. Brand resonance consists of four main aspects i.e., a sense of community, behavioral loyalty, active engagement, and attitudinal attachment (Husain et al., 2022). In order to develop brand resonance, consumers have to use the products of the brands more frequently and pay high attention to the information related to the brand to form a strong psychological engagement with the brand (Lithopoulos et al., 2021).

Customer affective commitment is developed as a result of the passionate connection of the customers with the brand that demonstrates personal identifications (Farid and Niu, 2021). Both mutual value and identification are closely related to affective commitment. Khraiwish et al. (2022) opined that affective commitment is deeply rooted in the attachment of the customers with the brand. Putting differently, the emotional attachment of the brand is also known as affective commitment. The consumers who are genuinely committed to the brand have an emotional attachment to the brand, thus the affective commitment is higher among these customers (Iglesias et al., 2019). In the literature, affective commitment has two dominant co-dimensions, i.e., continuance commitment and normative commitment. Both of these dimensions strongly impact the attitudes of the consumers. When customers experienced high affective commitment, they remain attached to the brand (Jaiswal and Dhar, 2016). Customers who have a high affective commitment not only become loyal to the brand but also tend to show repurchase behavior. Research shows that affective commitment of the consumers toward a brand is established as a result of high service or product value. Moreover, developing affective commitment require time as it builds over time. However, affective commitment has a long-term effect on the brands’ performance (Rather et al., 2019).

Wang et al. (2008) examined the factors of customer-based with product-market outcome approaches with brand resonance and quality perception as mediators. The authors suggested that future studies must analyze the determinants of brand equity such as perceived value in the existing model. Moreover, only a handful of studies have empirically analyzed the relationship between perceived value and customer-based brand equity. In addition, limited studies showed the factors affecting customer-based brand equity. Scare literature is available with regards to the mediating effect of brand resonance. Considering these shortfalls, the present study aimed to examine the role of product perceived value on customer-based brand equity with the mediating role of brand resonance. Moreover, Mbango (2018) analyzed the influence of customer satisfaction on repurchase intention with the mediation of affective commitment. The researchers argued that affective commitment must be studied with other variables related to new branded products. Therefore, the current study incorporated customer affective commitment as a mediator in the relationship between product perceived value and customer-based brand equity on branded products.

Based on the gap found in the literature, the present study postulated some objectives which are: to examine the impact of product perceived value on customer-based brand equity, to analyze the effect of product perception on brand resonance, and to determine the impact of product perceive value on customer affective commitment. The study has two mediating variables i.e., brand resonance and customer affective commitment, so the study has formulated the objective based on these constructs. The objectives are: (1) to investigate the mediating role of brand resonance in the relationship between product perceive value and customer-based brand equity and (2) to analyze the mediating role of customer affective commitment in the relationship between product perceive value and customer-based brand equity.

The current study also posited the research questions which have been addressed. The research questions are: what is the impact of product perceived value on customer-based brand equity? What is the effect of the product perceived on brand resonance? And what is the impact of product perceived value on customer affective commitment? The study has two mediating variables i.e., brand resonance and customer affective commitment, so the study has formulated the questions on the basis of these constructs. The research questions are: does brand resonance mediate the relationship between product perceived value and customer-based brand equity and does customer affective commitment mediate the relationship between product perceived value and customer-based brand equity?

## Review of Literature and Hypotheses Development

The study was conducted in China and the data was acquired from the customers who purchase branded products. This research intends to analyze the role of product perceived value and customer-based brand equity with the mediations of brand resonance and customer affective commitment. To address the objectives of the study, the study established the hypotheses which are based on the following theory.

### Marx’s Theory

Marx’s theory was developed by Marx in 1954 as a critique of the school of human relations. According to this perspective, organizations are not the rational system to perform work efficiently, rather organizational systems are power systems designed for the maximization of profits and control. Marx’s value-based perspective consists of two dimensions i.e., the sphere of production and the sphere of circulation. These spheres focus on the process of capital accumulation. According to Marx, there are three core values in the organizational context, i.e., use value, exchange value, and value. Use value is defined as the physical or other characteristics of a product that induce the demand for that product. It is the product utility. Exchange value highlights the monetary value given by the individuals in exchange for the product. It is the money from value. Value is referred to as the labor time embodied in the product. According to Marx’s perspective, value is socially necessary for provoking benefits for the organization (Bowman and Toms, 2010).

**FIGURE 1:**
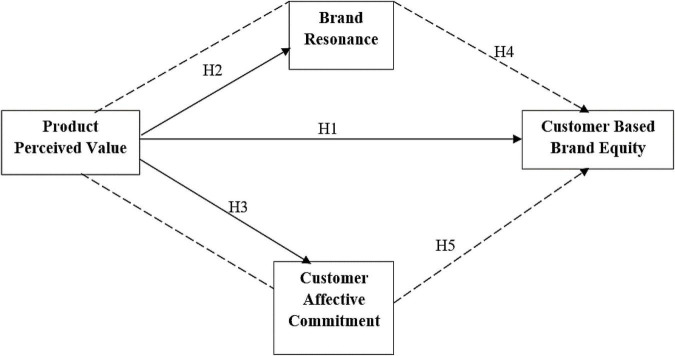
Theoretical framework.

Considering the framework of the study, product perceived value impacts customer-based brand equity, brand resonance, and customer affective commitment with the mediations of brand resonance and customer affective commitment. The relationship between the variables has been borrowed from Marx’s theory which points out that value are a significant facet of building an organization. The value-based perspective highlights the importance of value provided by the organization either use value, exchange value, or value. This theory is deeply rooted in the framework of the study (see Figure 1).

### Relationship Between Product Perceived Value and Customer-Based Brand Equity

Perceived value is the key facet and is regarded as an important factor of most customer-based brand equity frameworks (Surücü et al., 2019). Product perceived value mainly focuses on the assessment of the customer with regards to the utility of the brand on the basis of their perceptions of what they have received in terms of satisfaction and quality and what they have given in terms of money and other non-monetary costs. Perceived value is considered a significant aspect that leads toward customer-based brand equity (Wu et al., 2020). Perceived value includes experiential, functional, and symbolic attributes and functions. Moreover, scholars argued that perceive value includes perceived quality in such a way that when perceived quality is high, consumers develop high perceived value (Kim et al., 2021). Therefore, perceived quality is included in the judgment of perceived value.

Perceived value plays a significant role in building brand equity because customers need high value from the products. Customer-based brand equity consists of five major determinants, i.e., value, commitment, trust, social image, and performance. These determinants contribute greatly to developing customer-based brand equity. For example, Iglesias et al. (2019) undertook a study to examine the role of customer satisfaction, commitment, and brand experience on customer-based brand equity and found that these factors positively affect customer-based brand equity. Perceived value and customer-based brand equity are closely related because one of the dimensions of customer-based brand equity is product value (Dian et al., 2021). The product value offered to the customers by the brands can either increase or decrease the brand equity such that if the product value is high, brand equity will increase (Armawan, 2021). From the point of view of the customers, brand equity is the value offered to the target customers (Gallart-Camahort et al., 2021). Aaker’s model highlights the brand value and brand equity to measure and modularizes the overall brand equity. This model also suggests that product value is a significant component of brand equity and these constructs are gelled together (Ertemel and Civelek, 2020). Customer-based brand equity (CBBE) significantly contributes to the overall success of the brand. Ruan et al. (2020) opined that customer-based brand equity is developed as a result of perceived value because when the products offer high value to the customer, the brand equity increases. Moreover, studies also pointed out the fact that brand equity is an intangible factor that is based on the judgment and measurement of the perceived value (DAM, 2020). Moreover, perceived value can be explained as the difference between the total product value and total product cost. The total product value is the benefits acquired by the customers from the product and the total product cost is the energy time and money given by the customer during the purchase. If the total product value is higher than the total product cost then the product’s perceived value is high (Lee et al., 2020).

Few studies have been conducted to understand how perceived value impacts customer-based brand equity. For example, Yan and Yan (2019) studied the influence of customer perceived value on brand equity. The results showed that all three types of values (i.e., emotional value, functional value, and cost) have a positive effect on brand equity among customers in China. Jahanzeb et al. (2013) also examine the impact of service quality on brand equity with the mediations of perceived value and corporate credibility. The findings revealed that service quality positively influences brand equity and both perceived value and corporate credibility mediate the relationship. These findings showed that perceived value has a positive association with brand equity. Although few studies have examined the relationship between product perceived value and customer-based brand equity, still this relationship is not extensively tested and this further validation is required. Based on the above discussion, the following hypothesis is formulated:

***H*_1_.** *Product Perceived Value has a positive association with Customer-Based Brand Equity.*

### Relationship Between Product Perceived Value and Brand Resonance

(Keller, 2021) explained that brand resonance in terms of the association between the brand and its customers that brand resonance develops when the consumers sense the brand and establish emotional resonance with the brand. The four components of brand resonance are behavioral loyalty, attachment, sense of community, and active engagement. Loyalty indicates the value received by the customers which in turn develops customer buying behavior (Duman et al., 2018). This signifies that value is deeply associated with brand resonance. In addition, the relationship between perceived value and brand resonance can be explained through customer experience. The brand experience emphasizes the depth of the psychological bond which is built through the high perceived value of the product (Cheng et al., 2019). A pleasant experience creates brand awareness and enables the consumers to make a purchase decision and develop brand resonance.

Perceived value influences customer satisfaction such that when the customers receive high value from the product, they get satisfied. This satisfaction then leads to brand resonance. In this regard, (Jang et al., 2021) claimed that perceived value maximizes the satisfaction of the customers which in turn helps to build brand resonance. Moreover, (Khan et al., 2022) empirically tested the relationship between brand relationship quality and utility value on brand resonance using social exchange theory among Halal food consumers. The results confirmed that utility value has a positive association with brand resonance and brand relationship quality mediated the relationship. Moreover, from the consumer’s perspective, product value increases due to product utilization which leads to better performance of the product (Hwang et al., 2018). A study conducted on product quality and brand loyalty showed that higher quality leads to high brand loyalty (Husain et al., 2022). It indicates that utility value positively impacts brand loyalty and perceived value leads to long term bond of the customer with the brand. Thus, perceived value has a strong association with brand loyalty which is one of the dimensions of brand resonance.

The value offered to the customer builds brand image and reputation which consequently impacts brand resonance (Wang and Lin, 2021). The customers also require brand knowledge so that they can relate to the brand and understand what the brand offers. High-quality products offered to the customer develop high perceive value among the customers because quality matters to a greater extent when it comes to purchasing decisions (Lithopoulos et al., 2021). Additionally, brand resonance is particularly associated it how the consumers relate to the brand. If the value of the products is high, only then the consumers can relate and develop a positive perception of the brand. Only a few studies have examined the relationship between perceived value and brand resonance. For example, (Wang and Lin, 2021) examined the relationship between experiential value, celebrity endorsement, and brand resonance. The study confirmed that experiential value is associated with brand resonance through celebrity endorsement. Very scarce literature is available that explained the relationship between product perceived value and brand resonance. There is a need to analyze this association. Therefore, the present study aimed to address this gap in the literature, and posited the following hypothesis:

***H*_2_.** *Product Perceived Value has a positive impact on Brand Resonance.*

### Relationship Between Product Perceived Value and Customer Affective Commitment

Customers make a purchase decision on the basis of their evaluation in terms of how good their experience was with the brand (Gallart-Camahort et al., 2021). For example, if the product’s perceived value was high, they regard it as a pleasurable and satisfactory experience. In addition, when the experience of the customers with the product outperforms their experience, they perceive that product to have high value (Farid and Niu, 2021). Customers build a positive attitude toward the branded product if the product performance is higher than expected. This attitude enables the customers to purchase branded products (Khraiwish et al., 2022). Moreover, customers provide their judgment of the product based on the overall quality and the value obtained from buying the product. The products that have high quality and value are preferred by the customers as a result they show repurchase behavior (Husain et al., 2022). However, poor quality and low-value products of the brand are not only disliked by the customers but the customers tend to show a negative attitude toward the brand.

High product perceived value tends to contribute to the future commitment of the customers to the brands (Servera-Francés et al., 2019). Products with high value make the customer committed to the brand. Prior studies have shown that evaluation of the customers regarding product value based on their consumption experience leads to affective commitment (Kungumapriya and Malarmathi, 2018). A high level of product value is closely related to a strong relationship with the brand. In addition, customer affective commitment has a strong association with a brand’s performance (Molinillo et al., 2021). The marketing literature showed that customers with high perceived value show a high level of brand loyalty which in turn influences customer affective commitment (Wu et al., 2020). This commitment enables the consumers to provide positive recommendations and referrals about the brand. Therefore, it becomes significant for the brand to deliver high-quality and high-value products to the customers, so that the brand can be benefited in the long run.

Kungumapriya and Malarmathi (2018) opined that affective commitment is developed as a result of brand loyalty because loyalty has been conceptualized as the strong commitment of the customers to repurchase the product. According to Servera-Francés et al. (2019), customers develop a positive attitude, such as affective commitment and brand loyalty, toward a particular brand if the brand provides high-value products. The relationship between product perceived value and customer affective commitment has not been widely explored. However, few studies have linked these variables through a third construct. For example, Roy et al. (2022) investigated the influence of customer experience and customer affective commitment among Australian retailers. The authors claimed that customer experience is positively associated with affective commitment and this experience gets pleasant with high perceived value. Similarly, Itani et al. (2019) analyzed the association of perceived value and product quality with customer engagement. The findings confirmed that perceived value and product quality have a significant relationship with customer engagement. These results indicate that perceived value is also positively associated with customer affective commitment. Based on the above discussion, the following hypothesis has been proposed:

***H*_3_.** *Product Perceived Value has a positive impact on Customer Affective Commitment.*

### Mediating Role of Brand Resonance

Brand resonance is referred to as the relationship between the brand and the customer that includes the willingness of the customer to purchase or recommend the brand to others. The perception of the customers about the brand is built through their experiences and learning about the brand and what value they offer to the customers (Jang et al., 2021). Therefore, brands add value to the products by delivering meaning, and customers like brands as they package the meaning (Cheng et al., 2019). Product value significantly impacts brand resonance because when the customer receives highly valued products, they can relate to the brand. Put differently, brands can attract customers by providing products with a high value which leads to brand equity (Kang et al., 2021). Additionally, Jang et al. (2021) claimed that perceived value maximizes the satisfaction of the customers which in turn helps to build brand resonance. These studies justify that brand resonance can be a mediator in the relationship between perceived value and brand equity.

One of the core dimensions of brand equity is brand loyalty, and brand loyalty is built when the customer can relate to or resonate with the brand (Husain et al., 2022). This resonance is deeply linked with perceived value because customer needs are fulfilled when they receive high valued products (Lithopoulos et al., 2021). Scholars argued that perceived value has a positive relationship with brand resonance and customer-based brand equity; therefore, brands that want to enhance brand resonance and CBBE must deliver high-value products (Kang et al., 2021). Brand resonance can help predict brand equity, future revenues, and firm value in competitive business markets. Brands win customers because they develop a deep relationship with the customers through different factors. In a sense, to develop this relationship brands deliver products that exceed the expectations of the customers (Rather et al., 2019). Branding marketing is deeply rooted in what the brand offers to the customers and how the customers perceive those products.

The mediating role of brand resonance has been explored in a few studies only. For instance, Khan et al. (2022) examined the mediating role of brand resonance in the relationship between quality and utility value among food consumers. The results confirmed that brand resonance is a significant mediator between quality and utility value. The researchers discussed that quality products enhance brand resonance which consequently maximizes utility value. A recent study by Duman et al. (2018) also determined the influence of affective factors on brand equity with the mediation of brand resonance among Turkish visitors. Duman et al. (2018) discussed based on the results obtained that affective factors significantly impact brand equity and brand resonance fully mediated the relationship. Husain et al. (2022) empirically tested the influence of brand experience on brand trust with the mediating effect of brand resonance. This study also showed that brand resonance mediated the relationship between brand experience and brand trust. On the basis of the above discussion and past literature, the author developed the hypothesis mentioned below:

***H*_4_.** *Brand Resonance positively mediates the relationship between Product Perceived Value and Customer-Based Brand Equity.*

### Mediating Role of Customer Affective Commitment

In the marketing literature, customer affective commitment plays a key role as it is a major determinant of influencing the brands (Syed and Shanmugam, 2021). Scholars argued that customer affective commitment is directly related to customer-based brand equity (Zang et al., 2021). For example, Poushneh et al. (2019) found that affective commitment reduces the switching behavior and prevents the customers to search for alternative brands (DAM, 2020). The switching behavior can be decreased with the high perceived value gained by the customer after purchasing the products from a particular brand (Armawan, 2021). Customers with low switching intentions show high commitment to the brand, thus enhancing brand equity. This traditional understanding of affective commitment as a behavior for the repurchase of a product constitutes brand equity. Customers call for high valued products and this perceived value then leads to affective commitment. Putting differently, customers build a positive attitude toward the branded product if the product performance is higher than expected.

More recently, scholars recognize affective commitment as a significant factor for the brands as it induces customer desire to recommend the brand to peers (Farid and Niu, 2021). The preference of the customer for one brand over others and affective commitment leads to higher brand equity and brand success. Scholars also argued that affective commitment is one of the dimensions of brand equity (Iglesias et al., 2019). Moreover, academicians also proposed that affective commitment and attachment with the brand are dimensions of customer-based brand equity. Brands consider commitment as an internal brand strength that can induce brand equity (Slack et al., 2020). Furthermore, brand equity is conceptualized as a measure of the strength of customer commitment to a brand. In this regard, Wang et al. (2008) claimed that affective commitment can be developed in customers with high product perceived value.

Among the scant literature available relating to customer affective commitment as a mediator, Rather et al. (2019) found that customer affective commitment mediated the relationship between customer word-of-mouth and customer involvement. Jeon and Yoo (2021) also examined the association of perceived value and customer engagement through the mediation of affective commitment. Based on the results of the study, the authors concluded that perceived value significantly impacts customer engagement and affective commitment, and affective commitment facilities the relationship among the customer of the hotel industry. Finally, Syed and Shanmugam (2021) analyzed the influence of corporate social responsibility on word-of-mouth with the mediating effects of affective commitment and customer trust. The empirical evidence revealed that affective commitment and customer trust fully mediated the relationship between corporate social and word-of-mouth. In line with the scant literature and aiming to gain further insights into the mediating role of affective commitment in the relationship between product perceived value and customer-based brand equity, the author proposed that:

***H*_5_.** *Customer Affective Commitment positively mediates the relationship between Product Perceived Value and Customer-Based Brand Equity.*

Based on the above discussion and the literature, the authors developed the following conceptual framework (see Figure 1):

## Research Methods

### Study Design

This study aims to identify the effect of product perceived value on customer-based brand equity through the mediating role of brand resources and customer affective commitment. Hence, the present study data was gathered from consumers of various branded products in China. In this regard, the authors visited different brand outlets to seek permission for data collection from the customer. For this aim, they met with outlet managers, conveyed the complete data collection objective, and assured them that data would be collected only for educational purposes. The authors also guided the managers regarding the importance of the practical implication of the study for whom data would be collected. So, they also assured them that the practical implication would be shared upon their request. Hence, managers showed a positive response and were permitted to collect data.

The authors developed self-administrated questionnaires based on a cover letter for data collection. This cover letter conveyed the whole objective of data collection to the participants as this study helps the organizations ensure better services and products based on customers’ views. Moreover, through the cover letter, it was also conveyed that no answers are right and wrong as their true answers would help in generating natural outcomes for this study. Hence, this step boosts the confidence of the customers to get as natural as possible responses. The questionnaire was first developed in English and then translated into Chinese as English was not common in China for speaking and reading. Hence, the authors developed dual-language questionnaires to ease the customers’ understanding. Translation of questionnaires was completed under the guidance of senior researchers. As per their guidance, the author also collected sample base data from students on dual language questionnaires for language proficiency examination. In this way, all errors and corrections were made, and the senior researchers approved finalized questionnaire.

The authors adopted a convenient sampling technique for data collection from customers. They took two weeks for data collection and personally visited different brand outlets and gave approximately 8 h a day to collect data. They also requested all the visitors and customers of different outlets for questionnaire filling and offers soft drinks to customers to fill out the questionnaires. In this way, the authors targeted 350 customers and got back 350 questionnaires. After confirming their completeness and validness, they finalized 310 responses for data analysis. Hence, this study’s empirical analyses are based on a 310 sample size.

### Measures

The present study participants’ responses were measured based on five points Likert scale. This scale has five numbers where 1 represents “strongly disagree,” 2 represents “disagree,” 3 represents “neutral,” 4 represents “agree,” and 5 represents “strongly agree.” This study assessed data from previously validated items. The construct perceived product value was measured with five items scale adapted from Agarwal and Teas (2001). The construct brand resource was measured with six items scale adapted from Wang et al. (2008). The construct of customer affective commitment was measured with a five-items scale adapted from Dean (2007). The construct customer-based brand equity was measured with four items scale adapted from Boonghee et al. (2000). All variables items are presented in Appendix 1.

## Results

### Assessment of Measurement and Structural Model

The present study applied the variance-based partial least squares structural equation modeling (PLS-SEM) technique instead of other co-variance-based techniques such as AMOS. The basic purpose behind this selection is the effectiveness of PLS-SEM for both types of studies (confirmatory and exploratory) (Hair et al., 2011). Structural equation modeling (SEM) consists of two different types, which include covariance-based (CB-SEM) and PLS-SEM. The key difference in both methods is that CB-SEM is considered for theory acceptance and rejection, while PLS-SEM is considered for advancing and developing the theories (Avotra et al., 2021; Nawaz et al., 2021; Yingfei et al., 2021). PLS-SEM is an appropriate approach for complex and multi-orders-based models and needs no specific data normality conditions. PLS-SEM is also useful for evaluating small data sets (Hair et al., 2016). Hence, the present study considers the PLS-SEM method for empirical data analyses using Smart PLS 3.3.3 software. The outcomes of PLS-SEM-based analysis are evaluated in two stages, including model measurement and structural model evaluation. The measurement model stage assesses the reliability and validity of the constructs, whereas the structural model analyzes the relationship between the proposed hypotheses. The acceptance or rejection of a hypothesis is determined through the t statistic and *p* values.

The results of model measurement consist of two parts: model reliability and validity. The present study considered the values of “Cronbach’s alpha, roh-A, composite reliability, and average variance extract (AVE)” to approve the model’s reliability (Hair et al., 2016), and all values are presented in Table 1. The values of Cronbach’s alpha are accepted if they are greater than 0.7 (Hair et al., 2019b). In the same way, the composite reliability value should also be greater than 0.7. The Cronbach’s alpha values of models’ constructs (brand resonance, customer affective commitment, customer-based brand equity, and product perceived value) are 0.895, 0.872, 0.849, 0.884 and the composite reliability values of models’ constructs are 0.920, 0.907, 0.896, and 0.915, respectively. All values of Cronbach’s alpha and composite reliability are according to acceptable criteria, which confirm the model’s reliability in the present study. The values of roh-A reliability (0.899, 0.874, 0.863, 0.886) are also according to acceptable criteria (Hair et al., 2019b). The average variance extract (AVE) values greater than 0.5 are considered appropriate for the model’s convergent validity (Hair et al., 2011). The Table 1 illustrates that the AVE values (0.658, 0.662, 0.684, and 0.684) are according to acceptable criteria.

**TABLE 1:** Reliability and convergent validity of the study constructs.

Construct	Item	Outer loadings	VIF	Alpha	roh-A	Composite reliability	AVE
BR	BR1	0.847	2.633	0.895	0.899	0.920	0.658
	BR2	0.803	2.227				
	BR3	0.842	2.611				
	BR4	0.821	2.630				
	BR5	0.843	2.803				
	BR6	0.699	1.502				
CAC	CAC1	0.853	2.387	0.872	0.874	0.907	0.662
	CAC2	0.773	1.899				
	CAC3	0.814	2.004				
	CAC4	0.802	1.986				
	CAC5	0.824	2.012				
CBBE	CBBE1	0.838	2.362	0.849	0.863	0.896	0.684
	CBBE2	0.850	3.287				
	CBBE3	0.867	3.488				
	CBBE4	0.749	1.294				
PPV	PPV1	0.838	2.223	0.884	0.886	0.915	0.684
	PPV2	0.790	1.893				
	PPV3	0.835	2.260				
	PPV4	0.878	2.949				
	PPV5	0.792	2.078				

*PPV = Product Perceived Value, CBBE = Customer-Based Brand Equity, BR = Brand Resonance, CAC = Customer Affective Commitment.*

**FIGURE 2:**
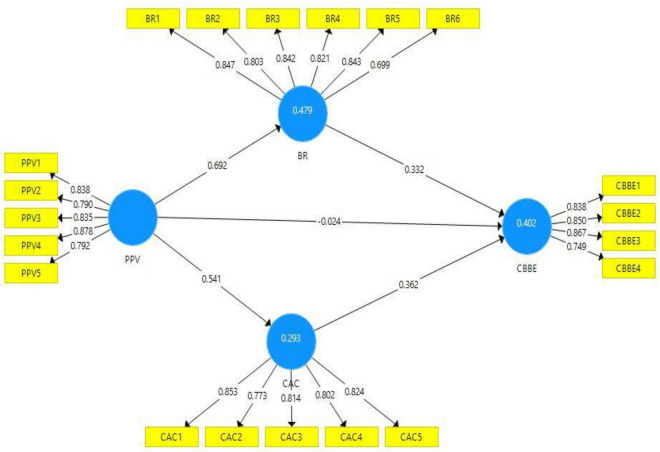
Output of measurement model. PPV = Product Perceived Value, CBBE = Customer-Based Brand Equity, BR = Brand Resonance, AC = Affective Commitment.

Table 1 reported that the current study model is based on 20 items of the four variables. According to acceptable criteria, the outer loading values greater than or equal to 0.7 are considered reliable for the model’s validity (Hair et al., 2019b). Figure 2 depicts that the outer loading values of all items are according to the acceptance criteria. One item (BR6) shows the outer loading value of 0.699, which is below 0.7 but retained because this item did not affect the AVE value. The variance inflation factor (VIF) values are also displayed in Table 1. The VIF values are evaluated to verify the collinearity issues in the model. The model is considered free from the collinearity problems if the VIF values are reported below 0.5 (Hair et al., 2019b). According to the outcomes presented in Table 1, the VIF values are less than 0.5, such as the variable “customer-based brand equity” item CBBE-3 has the highest VIF value (3.488). Hence, it is proved that there are no collinearity issues in the current study model (Hair et al., 2016).

The R^2^ values are measured to describe the model’s strength, such as the values of latent variables exceeding or near 0.5, which indicates moderate strength of the model, and the values near 0.25 show weak model strength (Hair et al., 2017). The R^2^ values of endogenous variables of the present study’ model (Brand Resonance, Affective Commitment, Customer-Based Brand Equity) are 0.479, 0.293, and 0.402, respectively, which shows moderate model strength (Hair et al., 2019a). The cross-validated redundancy (Q^2^) values of the model are considered significant if they are larger than zero (Hair et al., 2017). The Q^2^ values of all latent variables of the current study are greater than zero, which validates the significance of the model.

The two well-known approaches, namely, Fornell–Larcker criterion and Heterotrait–Monotrait (HTMT) ratios, are used to evaluate the discriminant validity of the current study (Hair et al., 2011). The Fornell-Larcker criterion is evaluated by taking the square roots of AVE values of model constructs (Hair et al., 2014). The Forenell-Larcker criterion values of variables are presented in Table 2. The values under the Forenell-Larcker criterion are accepted if the upper side first value of each column is highest than their below values. Table 2 shows that all values of the Forenell-Larcker criterion are as per the accepted criteria. Thus, it is confirmed that discriminant validity based on the Fornell-Larcker criterion has been achieved in this study model. In addition, according to the given criteria, the HTMT values of all constructs should be less than 0.85; however, values greater than 0.90 are also considerable (Nawaz et al., 2019; An et al., 2021; Xiaolong et al., 2021). According to the outcomes of the present study, the HTMT values of constructs are less than 0.85, which confirmed that discriminant validity in the present study’s model has been established (Table 3).

**TABLE 2:** Discriminant validity (Fornell-Larker-1981 Criteria).

Construct	BF	CAC	CBBE	PPV
BR	***0.811***			
CAC	0.748	***0.814***		
CBBE	0.587	0.598	***0.827***	
PPV	0.692	0.541	0.402	***0.827***

*PPV = Product Perceived Value, CBBE = Customer-Based Brand Equity, BR = Brand Resonance, CAC = Customer Affective Commitment.*

**TABLE 3:** Discriminant validity (HTMT).

Construct	BF	CAC	CBBE	PPV
BR	–	**–**	**–**	**–**
CAC	0.846	–	**–**	**–**
CBBE	0.640	0.657	–	–
PPV	0.778	0.616	0.448	**–**

*PPV = Product Perceived Value, CBBE = Customer-Based Brand Equity, BR = Brand Resonance, CAC = Customer Affective Commitment.*

## Hypotheses Analysis

### Direct Effect

The empirical investigation of the current study was accompanied by using a bootstrapping approach through 5000 samples with replacements to measure the significance level. The direct, indirect, and total paths are presented in Table 4. The present study considered the “*t*” values and “*p*” values of statistics for the acceptance or rejection of the hypotheses. The current study hypothesis results are shown in Table 5. According to hypothesis 1, product perceived value positively impacts customer-based brand equity; however, the outcomes (*t* = 0.350, *p* = 0.726) depicted product perceived value does not positively impact customer-based brand equity. Hence, the first hypothesis of this study is rejected. The outcomes (*t* = 12.876, *p* = 0.000) of hypothesis 2 confirmed that the product’s perceived value positively impacts brand resonance, which means the second hypothesis of the present study is accepted. In addition, the beta value of hypothesis 2 revealed that one unit change in the independent variable (Product Perceived Value) would result in 0.692 changes in the dependent variable (Brand Resonance). According to the results (t = 6.714, p = 0.000) of the third hypothesis, product perceived value positively impacts customer affective commitment, confirming that the second assumption of the present study is accepted. In addition, the beta value of H3 showed that one unit change in the independent variable (Product Perceived Value) would result in 0.541 changes in the dependent variable (Customer Affective Commitment).

**TABLE 4:** Direct, indirect and total path estimates.

Direct path	Beta	SD	T	P
BR -> CBBE	0.332	0.078	4.254	**0.000**
CAC -> CBBE	0.362	0.075	4.865	**0.000**
PPPV -> BR	0.692	0.054	12.876	**0.000**
PPPV -> CAC	0.541	0.081	6.714	**0.000**
PPV -> CBBE	−0.024	0.068	0.350	**0.726**

**Indirect path**	**Beta**	**SD**	**T**	**P**

PPV -> BR -> CBBE	0.230	0.059	3.894	**0.000**
PPV -> CAC -> CBBE	0.196	0.056	3.490	**0.000**

**Total path**	**Beta**	**SD**	**T**	**P**

BR -> CBBE	0.332	0.078	4.254	**0.000**
CAC -> CBBE	0.362	0.075	4.865	**0.000**
PPPV -> BR	0.692	0.054	12.876	**0.000**
PPPV -> CAC	0.541	0.081	6.714	**0.000**
PPV -> CBBE	0.402	0.083	4.835	**0.000**

*PPV = Product Perceived Value, CBBE = Customer-Based Brand Equity, BR = Brand Resonance, CAC = Customer Affective Commitment.*

**TABLE 5:** Hypotheses testing (Direct Effect).

Hypotheses		Coefficient (Beta)	S.D	*T*	*P*	Status
H1	PPV -> CBBE	−0.024	0.068	0.350	0.726	Not Supported
H2	PPV -> BR	0.692	0.054	12.876	0.000	Supported
H3	PPV -> CAC	0.541	0.081	6.714	0.000	Supported

*PPV = Product Perceived Value, CBBE = Customer-Based Brand Equity, BR = Brand Resonance, CAC = Customer Affective Commitment.*

### Indirect Effect/Mediation

**FIGURE 3:**
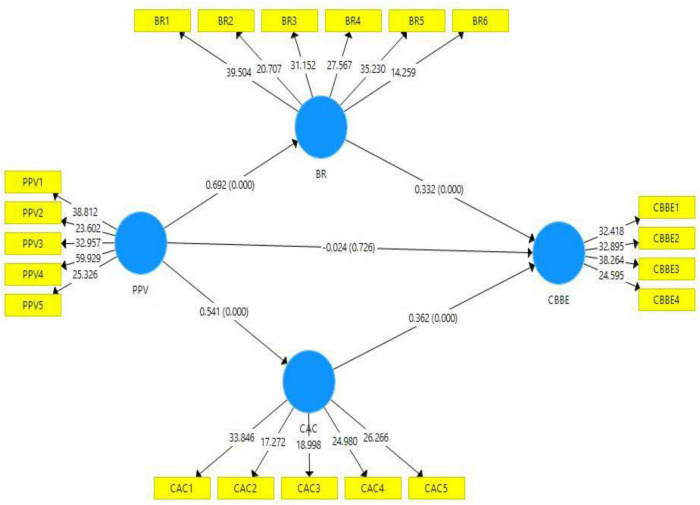
Structural model bootstrapping. PPV = Product Perceived Value, CBBE = Customer-Based Brand Equity, BR = Brand Resonance, AC = Affective Commitment.

The present study also considered the mediating role of brand resonance and customer affective commitment between product perceived value and customer-based brand equity, respectively (Figure 3). For the empirical investigation of brand resonance and customer affective commitment as mediators, this study assumes hypotheses 4 and 5 (Table 6). The present study used the variance accounted for (VAF) approach to indicate the mediation level. The values of VAF greater than 80% show full mediation, values between 20 and 80% show partial mediation, and values less than 20%. The results of hypothesis 4 revealed that H4 is accepted, and the VAF value (57.21%) shows partial mediation. According to the outcomes, hypothesis 5 of the present study is also accepted, and the VAF value (48.75%) shows partial mediation.

**TABLE 6:** Hypotheses testing (Mediation).

	Paths	Direct effect (*t*-value)	Indirect effect (*t*-value)	Total effect	VAF%	Interpretation	Results
H4	PPV-> BR-> CBBE	−0.024 (0.350)	0.230 (3.894)	0.402 (4.835)	57.21	Partial Mediation	Supported
H5	PPV-> CAC-> CBBE	−0.024 (0.350)	0.196 (3.490)	0.402 (4.835)	48.75	Partial Mediation	Supported

*PPV = Product Perceived Value, CBBE = Customer-Based Brand Equity, BR = Brand Resonance, CAC = Customer Affective Commitment.*

## Discussion

In this turbulent era of competition, organizations seek ways to enhance performance and be more competitive in markets. Boukis and Christodoulides (2020) point out that higher brand equity is a constructive indicator of future success and the long-term sustainability of the firms. Customer-based brand equity is a phenomenon that has become the point of attention from various researchers and scholars worldwide (Yan and Yan, 2019). Studies have been initiated to investigate how customer-based brand equity can be developed and enhanced. However, there is a dearth of knowledge concerning the various antecedents and predictors of brand-based equity. Based on Marx’s theory, the present study attempts to determine the role of product perceived value on customer-based brand equity, brand resonance and customer affective commitment, respectively. Moreover, this study also tries to determine the mediating roles of brand resonance and customer affective commitment in the relationship between product perceived value and customer-based brand equity, respectively.

The outcomes of the present study depicted that hypothesis 1 (product perceived value has a positive impact on customer-based brand equity) is not accepted, which means that the product perceived value does not have a positive association with customer-based brand equity online. However, these results are not consistent with prior studies (Surücü et al., 2019; Yan and Yan, 2019), as these studies claimed that perceived value is the key facet and is regarded as an important factor in most customer-based brand equity frameworks. Moreover, the present study’s findings confirmed that product perceived value positively impacts brand resonance and customer affective commitment, respectively, which means that hypotheses 2 and 3 are accepted. These findings are consistent with the findings of Roy et al. (2022), as they acknowledged that customers’ brand experience has a critical role in building their affective commitment, and this experience gets pleasant with high perceived value. Similarly, these findings are also confirmed by Kungumapriya and Malarmathi (2018). From their point of view, affective commitment is developed due to brand loyalty because loyalty has been conceptualized as the strong commitment of the customers to repurchase the product. The reason is the benefits associated with the product perceived value to the customers of the brands.

The findings of hypothesis 4 revealed that brand resonance positively mediates the relationship between product perceived value and customer-based brand equity. Khan et al. (2022) also confirmed that brand resonance is a significant mediator between quality and utility value. Additionally, the results are consistent with the findings obtained by Husain et al. (2022). They empirically tested the influence of brand experience on brand trust with the mediating effect of brand resonance and found that brand resonance mediated the relationship between brand experience and brand trust. Brand resonance is developed when the brand provides high valued products to the customers, consequently impacting customer-based brand equity.

The findings of hypothesis 5 confirmed that customer affective commitment positively mediates the relationship between product perceived value and customer-based brand equity. Jeon and Yoo (2021) also found that customer affective commitment plays a constructive role in strengthening the relationship between product perceived value and customer-based brand equity. Binu Raj (2021) concluded that perceived value significantly impacts customer engagement, and affective commitment and affective commitment facilitate the relationship among the customer of the hotel industry. Similarly, Slack et al. (2020) also revealed that brands consider commitment as an internal brand strength that can induce brand equity. Affective commitment is developed as a result of perceived value and this, in turn, influences customer-based brand equity.

## Theoretical and Practical Implications

This research study contributes in the body of literature in several significant ways. Existing research depicts that there are limited literature exists which explore the predictors of consumer-based brand equity (Mbango, 2018). Moreover, only a handful of studies have empirically analyzed the relationship between perceived value and customer-based brand equity. In addition, limited studies showed the factors affecting customer-based brand equity. Khan et al. (2022) suggest that future studies must analyze the predictors of consumer-based brand equity (Ertemel and Civelek, 2020). Thus this study extends the limited literature of consumer-based brand equity by exploring some key predictors of consumer-based brand equity. In addition to this, literature reveals that very scare literature exists which explore the mediation effect of brand resonance. Considering these shortfalls, the present study aimed to examine the role of product perceived value on customer-based brand equity with the mediating role of brand resonance. Moreover, there was a paucity of research with regards to how brand resonance and affective commitment mediate the relationship between perceived product value and customer-based brand equity (Iglesias et al., 2019). This study successfully bridged this gap by establishing the indirect effects of brand resonance and affective commitment. It was established that both brand resonance and affective commitment are important factors that indirectly influence the development of customer-based brand equity.

Moreover, analyzed the influence of customer satisfaction on repurchase intention with the mediation of affective commitment. The researchers argued that affective commitment must be studied with other variables related to new branded products. Therefore, the current study incorporated customer affective commitment as a mediator in the relationship between product perceived value and customer-based brand equity on branded products.

The results of this study also yield some important practical implications. It becomes imperative for the branding and marketing managers of various brands to undertake efforts and implement strategies to enhance the perceived value of their product offerings. The enhancement of perceived value will lead to the development of positive behavioral outcomes such as an increase in customer-brand-based equity. Some of the ways in which product perceived value can be enhanced include: improving product design, effectively communicating product benefits, and executing effective promotional strategies. Moreover, the marketing managers and organizations should also focus on enhancing brand resonance and affective commitment of the customers toward the brand. To achieve this, the organization should focus on establishing brand awareness and developing strong relationships between the organization and its customer base. This can be achieved by providing exceptional customer service and by implementing high values of transparency and customer-centricity.

## Limitations and Recommendations

Like other studies of social sciences, the current study also has some limitations, which may motivate scholars to conduct the research in the future. The sample size of this study was small and hence the results may not be generalizable; future studies may enlarge the sample size to enhance the reliability of the results. This study is conducted on Chinese customers only; future studies may expand the scope to other regions to enhance the reliability of the results. Moreover, a cross-sectional design was adopted by the present study. Future researchers should adopt a longitudinal design by acquiring data over multiple time intervals to enhance the credibility of the results. This study analyzed the impact of product perceived value on customer-based brand equity through the mediating mechanisms of brand resonance and affective commitment. Future studies should introduce other mediating variables such as perceived brand image, customer engagement, and brand awareness to broaden the understanding of the various antecedents and predictors of customer-based brand equity.

## Conclusion

In this era of competition, firms are seeking ways to enhance performance and be more competitive in markets. Customer-based brand equity is a phenomenon that has become the point of attention for various researchers and scholars worldwide. Studies have been initiated to investigate how customer-based brand equity can be developed and enhanced. However, there is a dearth of knowledge concerning the various antecedents and predictors of brand-based equity. Based on Marx’s theory, the present study attempts to determine the role of product perceived value on customer-based brand equity, brand resonance and customer affective commitment, respectively. Moreover, this study also tries to determine the mediating roles of brand resonance and customer affective commitment in the relationship between product perceived value and customer-based brand equity, respectively. The present study’s findings acknowledge that product perceived value did not directly influence customer-based brand equity. However, results confirmed that product perceived value positively influences brand resonance and customer affective commitment. Furthermore, the outcomes of the present study also concluded that both brand resonance and affective commitment played a mediating role between product perceived value and customer-based brand equity, respectively.

## Data Availability Statement

The original contributions presented in this study are included in the article/supplementary material, further inquiries can be directed to the corresponding author/s.

## Ethics Statement

The studies involving human participants were reviewed and approved by Harbin Normal University, China. The patients/participants provided their written informed consent to participate in this study. The study was conducted in accordance with the Declaration of Helsinki.

## Author Contributions

GX conceived and designed the concept. XY collected the data. YQ wrote the manuscript. All authors contributed to the article and approved the submitted version.

## Conflict of Interest

The authors declare that the research was conducted in the absence of any commercial or financial relationships that could be construed as a potential conflict of interest.

## Publisher’s Note

All claims expressed in this article are solely those of the authors and do not necessarily represent those of their affiliated organizations, or those of the publisher, the editors and the reviewers. Any product that may be evaluated in this article, or claim that may be made by its manufacturer, is not guaranteed or endorsed by the publisher.

## Appendix

**APPENDIX 1:** Constructs and their items.

Items	Measures
Perceived Product Value	1. This product is a very good value for money. 2. At the price shown, this product is very economical. 3. I consider this product to be a good buy. 4. The price shown for this product is very acceptable. 5. This product appears to be a bargain.
Customer-based Brand Equity	1. It makes sense to buy X instead of any other brand, even if they are the same. 2. Even if another brand has same features as X, I would prefer to buy X. 3. If there is another brand as good as X, I prefer to buy X. 4. If another brand is not different from X in any way, it seems smarter to purchase X.
Affective Commitment	1. I really care about the fate of this company 2. I feel a great deal of loyalty to this company 3. I am willing to put in effort to help this company be successful 4. I feel a sense of belonging to this company 5. My relationship with this company is very important to me
Brand Resonance	1. I would like to buy this brand 2. I consider myself to be loyal to this brand 3. I am willing to recommend this brand to my friends 4. I am used to this brand 5. This brand would be my first choice 6. I will not buy other brands if this brand is available at the store
